# Pulmonary function changes in older adults with and without metabolic syndrome

**DOI:** 10.1038/s41598-021-96766-x

**Published:** 2021-08-30

**Authors:** Maysa Alves Rodrigues Brandao-Rangel, Renilson Moraes-Ferreira, Manoel Carneiro Oliveira-Junior, Alana Santos-Dias, André Luis Lacerda Bachi, Giovana Gabriela-Pereira, Simone de Oliveira Freitas, Amanda Cristina Araújo-Rosa, Luis Vicente Franco Oliveira, Claudio Ricardo Frison, Wagner Luiz do Prado, Raghavan Pillai Raju, P Babu Balagopal, Rodolfo P Vieira

**Affiliations:** 1 Post-Graduation Program in Sciences of Human Movement and Rehabilitation, Federal University of Sao Paulo (UNIFESP), Avenida Ana Costa 95, Santos, SP 11060-001 Brazil; 2 Brazilian Institute of Teaching and Research in Pulmonary and Exercise Immunology (IBEPIPE), Rua Pedro Ernesto 240, São José dos Campos, SP 12245-520 Brazil; 3 Department of Otorhinolaryngology, Federal University of São Paulo (UNIFESP), Rua Pedro de Toledo 947, São Paulo, 04039-032 Brazil; 4 Post-Graduation Program in Human Movement and Rehabilitation, UniEvangélica, Avenida Universitária Km 3,5, Anápolis, GO 75083-515 Brazil; 5 Kinesiology Department, California State University San Bernardino, San Bernardino, CA USA; 6 Department of Pharmacology and Toxicology, Medical College of Georgia, Augusta University, Augusta, GA USA; 7 Nemours Children’s Health Systems, Jacksonville, FL USA; 8 Mayo Clinic College of Medicine, Jacksonville, FL USA; 9 Mayo Clinic College of Medicine, Rochester, MN USA; 10 Post-Graduation Program in Bioengineering, Universidade Brasil, Rua Carolina Fonseca 235, São Paulo, SP 08230-030 Brazil

**Keywords:** Immunology, Biomarkers, Endocrinology

## Abstract

The low-grade inflammation associated with metabolic syndrome (MS) triggers functional and structural alterations in several organs. Whereas lung function impairment is well reported for older adult population, the effect of MS on functional and immunological responses in the lungs remains unclear. In this cross-sectional study we determined whether MS alters pulmonary function, and immunological responses in older adults with MS. The study sample consisted of older adults with MS (68 ± 3 years old; n = 77) and without MS (67 ± 3 years old; n = 77). Impulse oscillometry was used to evaluate airway and tissue resistance, and reactance. Biomarkers of inflammation and fibrosis were assessed in the blood and in breath condensate. The total resistance of the respiratory system (R5Hz; *p* < 0.009), and the resistance of the proximal (R20Hz; *p* < 0.001) and distal (R5Hz–R20Hz; *p* < 0.004) airways were higher in MS individuals compared to those without MS. Pro-inflammatory (leptin, IL-1beta, IL-8, *p* < 0.001; TNF-alpha, *p* < 0.04) and anti-inflammatory cytokines (adiponectin, IL-1ra, IL-10, *p* < 0.001), anti-fibrotic (relaxin 1, relaxin 3, Klotho, *p* < 0.001) and pro-fibrotic (VEGF, *p* < 0.001) factors were increased in sera and in breath condensate individuals with MS. The results show that MS adversely affect lung mechanics, function, and immunological response in older adults. The data offer a metabolic basis for the inflammaging of the lungs and suggest the lungs as a potential therapeutic target for controlling the immune response and delaying the onset of impaired lung function in older adults with MS.

## Introduction

Metabolic syndrome (MS) is characterized by the coexistence of at least three of the following clinical features: abdominal obesity (AO), hyperglycemia, hypertriglyceridemia, hypertension and low levels of high-density lipoprotein (HDL)^1^. MS is also associated with low-grade inflammation characterized by increased circulating levels of pro-inflammatory factors, such as interleukin (IL) -1beta, IL-8, tumor necrosis factor alpha (TNF-alpha), leptin, resistin as well as pro-fibrotic growth factors, such as vascular endothelial growth factor (VEGF) and transforming growth factor beta (TGF-beta)^2^. A heightened and chronic state of inflammation in older adults, frequently referred to as inflammaging, accelerates the biological aging process and exposes individuals to altered immune responses leading to immunosenescence. In individuals with MS compared to those without MS, inflammaging and immunosenescence are more pronounced and may induce structural and functional alterations in multiple organ systems, accelerating the overt manifestation of various diseases such as cardiovascular disease (CVD) and type II diabetes mellitus (T2DM)^3,4^. Additionally, the aging process itself entails obligatory changes in various body systems leading to drastic derangements including that in the respiratory system^5^. However, there is a paucity of information on the intrinsic changes in inflammatory status within the respiratory system of older adults with MS.

Although some controversy exists as to whether MS is a unique disease entity, its individual components have independently been associated with changes in pulmonary function and or lung diseases in humans^6^. The influence of MS on lung mechanics (indicating structural alterations) and pulmonary immune response, however, remains less clear, particularly in older adults. In fact, chronic respiratory diseases appear to be more frequent in individuals with morbid obesity and MS vs those without MS^7^. Previous studies have also reported correlations between systemic inflammation and reduced pulmonary function^8^. A recent pre-clinical study showed that obesity induced the development of a specific pro-inflammatory and pro-fibrotic lung phenotype, which might alter lung function^9^, suggesting the potential obesity-related alterations in lung function. In the present study, we tested the hypothesis that in older adults with MS, the pro-inflammatory and pro-fibrotic responses in the respiratory system may enhance the loss of pulmonary function and impaired lung mechanics.

## Methods

### Patient selection

Older adult women and men for the study were recruited in the Center for Social, Sports and Health Care for Older adults from the municipality of the city of São José dos Campos – SP, Brazil. World Health Organization (WHO) criteria for older adults defined as 60 years of age or older^10^ was used for recruitment. For inclusion in the study the participants should be able to perform spirometry evaluation (forced maneuver). Exclusion criteria included, (i) history of smoking, (ii) diagnosis of respiratory disease, (iii) chronic degenerative, autoimmune or neurological diseases, (iv) regular physical activity.

From a total 807 potential participants screened for the study, 77 (68 ± 3 years old; 26 men, 51 women) with MS and 77 (67 ± 3 years old; 21 men, 56 women) without MS were randomly selected for this study. The diagnosis of MS was performed according to the American Heart Association’s diagnostic criteria^1^. Briefly, individuals presenting at least three of the following characteristics were classified as with MS: abdominal obesity (waist circumference t ≥ 102 cm for men and ≥ 88 cm for women); hyperglycemia (hyperinsulinemia: top 25% of fasting insulin values from non-diabetic population), hypertriglyceridemia (triglycerides ≥ 1.7 mmol/L), hypertension (blood pressure > 130/85 mm Hg) and low levels of high-density lipoprotein (Low HDL cholesterol: < 1.03 mmol/L (male), < 1.3 mmol/L (female)^1^.

Informed consent was obtained from all subjects after the nature of the study had been explained. The present study and all procedures performed were approved by the ethical committee of University of Sao Paulo (53344616.6.0000.5511) and appropriate consents were obtained from the participants included in the study according to the national recommendations for clinical studies, in agreement with Declaration of Helsinki.

### Clinical, biochemical and anthropometric evaluation

All volunteers were systematically evaluated and followed by a geriatrician from older adults of the municipality of São José dos Campos. The age (years), body mass (Kg), height (m), body mass index (BMI), and waist circumference (cm), were measured as part of the clinical evaluation of the volunteers. The venous blood (5 mL) was collected from each subject using vacuum tubes and 25 µl of the total blood was immediately used for the whole blood hematology analysis. The remaining blood was centrifuged at 900 g, 4 °C, for 7 min and the serum was stored at – 86 °C until analysis. Biochemical measurements consisted of total cholesterol (Code-REF76), HDL cholesterol (Code-REF13) and triglycerides (Code-REF87) in the sera by using commercial colorimetric kits from Labtest (Lagoa Santa, MG, Brazil). The whole blood analysis (white and red cells) was performed using the automated hematology analyzer (Roche, Sysmex XS-800i, Europe GmbH, Germany). Table 1 summarizes the clinical, biochemical, and anthropometric characteristics of the volunteers. The serum was used for the quantification of inflammatory and fibrotic mediators by enzyme-linked immunosorbent assay (ELISA), by using SpectraMax i3 (Molecular Devices, USA).

**Table 1:** Clinical and anthropometric characteristics of older adults with and without MS (n = 77 each in the MS and ‘without MS’ groups).

Parameters	MS	Without MS	*P* value
Age (years)	66 (63–71)	68 (63–73)	0.2298
Men (n)	26	23	–
Women (n)	51	54	–
Weight (kg)	73 (66–88)	65 (59–71)	< 0.0001
Height (m)	1.58 (1.53–1.63)	1.58 (1.52–1.64)	0.6850
BMI (kg/m^2^)	29.43 (± 3.3)	25.89 (± 3.4)	0.0002
Systolic blood pressure (mmHg)	142.1 (± 1.4)	118.1(± 1.4)	0.0001
Diastolic blood pressure (mmHg)	95.4 (± 0.91)	74.4 (± 0.9)	0.0001
Waist circumference (cm)	92 (86–100)	86 (81–91)	0.0003
Total cholesterol (mg/dl)	116 (91.25–163.5)	155.5 (105.5–193.2)	0.0092
HDL cholesterol (mg/dl)	39.5 (36–45.7)	38 (35–46.7)	0.8322
Triglycerides (mg/dl)	231.5 (163.2–298.7)	115 (98–150)	< 0.0001
Total leukocytes (cells/mm^3^)	6.17 (4.55–7.14)	6.45 (4.7–7.0)	0.5501
Basophils (cells/mm^3^)	20 (10–30)	10 (10–20)	0.0086
Monocytes (cells/mm^3^)	434.5 (± 4.1)	318.42 (± 3.2)	0.0026
Eosinophils (cells/mm^3^)	135 (87.5–230)	130 (87.5–205)	0.7044
Lymphocytes (cells/mm^3^)	2.16 (1.63–2.88)	2.16 (1.63–2.88)	0.3169
Neutrophils (cells/mm^3^)	3.23 (2.35–3.94)	2.23 (1.30–3.56)	0.0027

*MS* metabolic syndrome. Mean (± Standard deviation), Median (interquartile interval). *BMI* body mass index, *HDL* high density lipoprotein, *LDL* low density lipoprotein.

### Breath condensate collection and analysis

The exhaled breath condensate (BC) was collected using the RT-Tube (Respiratory Research, USA) according to the manufacturer’s instructions. In brief, approximately 1–2 ml of BC was collected from each volunteer in 10–15 min and the samples were stored at – 86 °C until analysis. The BC was used for the quantification of inflammatory and fibrotic mediators by enzyme-linked immunosorbent assay (ELISA), using SpectraMax i3 (Molecular Devices, USA).

### Measurement of inflammatory and fibrotic mediators in serum and in breath condensate

Pro-inflammatory cytokines (IL-1beta, Biolegend 437006); (IL-8, R&D Systems DY208), (TNF-alpha, R&D Systems DY210), anti-inflammatory cytokines (IL-1ra, R&D Systems DY280) (IL-10, Biolegend 430603), pro-fibrotic (VEGF, R&D Systems DY293) and anti-fibrotic factors (relaxin 1, R&D Systems DY3257), (relaxin 3, R&D Systems DY3107), and (Klotho, R&D Systems DY5334) were evaluated by using ELISA DuoSet kits (R&D Systems, USA) or ELISA Max (Biolegend, USA), in serum and in breath condensate according to manufacturer’s instructions. The readings were performed using the multi-reader platform SpectraMax i3 (Molecular Devices, USA).

### Measurement of pulmonary function and mechanics

The lung function and mechanics were evaluated by using spirometry coupled to impulse oscillometer (Masterscreen Impulse Oscillometry – MS-IOS; Jaeger, Germany) using the American Thoracic Society (ATS) criteria^11^. The spirometric variables measured were: Forced vital capacity (FVC), forced expiratory volume in the first second (FEV1), the FEV1/FVC ratio, and the forced expiratory flow 25–75% (FEF25-75%)] using the reference values previously established for Brazilian population^12^. The lung mechanics were evaluated by impulse oscillometry (IOS), for the following parameters: R5Hz (total respiratory system resistance), R20Hz (resistance of proximal airways), R5Hz-R20Hz (resistance of distal airways), X5Hz (tissue reactance), RCentral (resistance of proximal tissue), and RPeripheral (resistance of distal tissue)^12^.

### Measurement of general and respiratory muscle strength

Hand grip strength, which represented the overall muscle strength was evaluated by using a hand grip dynamometer (Jamar, Sammons Preston Rolyan, Boilingbrook, IL, USA)^13^. The results were presented in kilogram (Kg). The respiratory muscle strength was evaluated by using a manovacuometer (MVD-300V.1.1 Microhard System, Globalmed, Porto Alegre, Brazil) by measuring the maximal inspiratory (MIP) and expiratory (MEP) pressure. The results were presented in cmH_2_O^14^.

### Statistical analysis

Data of clinical characteristics were summarized by individuals with MS and without MS. The quantitative variables were summarized by mean (SD) or median (interquartile interval [IQR], when appropriate). The normality of the data was analyzed by Shapiro–Wilk test and the correlation by Pearson test. Non-paired t-test or Mann–Whitney test was used to calculate significance and, *p* value of < 0.05 was considered significant. Categorical variables were summarized by numbers and percentages. Pearson correlation analysis was performed to examine the association of cytokines with pulmonary function. The GraphPad Prism 5.0 was used to perform the statistical analysis and build the graphs.

## Results

The study included equal number (n = 77 each) of participants with MS and without MS. Clinical, biochemical, and anthropometric characteristics of the subjects are shown in Table 1. Both groups were similar in age, height, total leukocytes, eosinophils, and lymphocytes. However, older adults with MS had significantly increased mean body weight, body mass index (BMI), systolic and diastolic blood pressure, waist circumference, total cholesterol, HDL cholesterol, triglycerides, basophils, monocytes, and neutrophils when compared to those without MS.

### Systemic inflammatory and fibrotic factors

**Figure 1:**
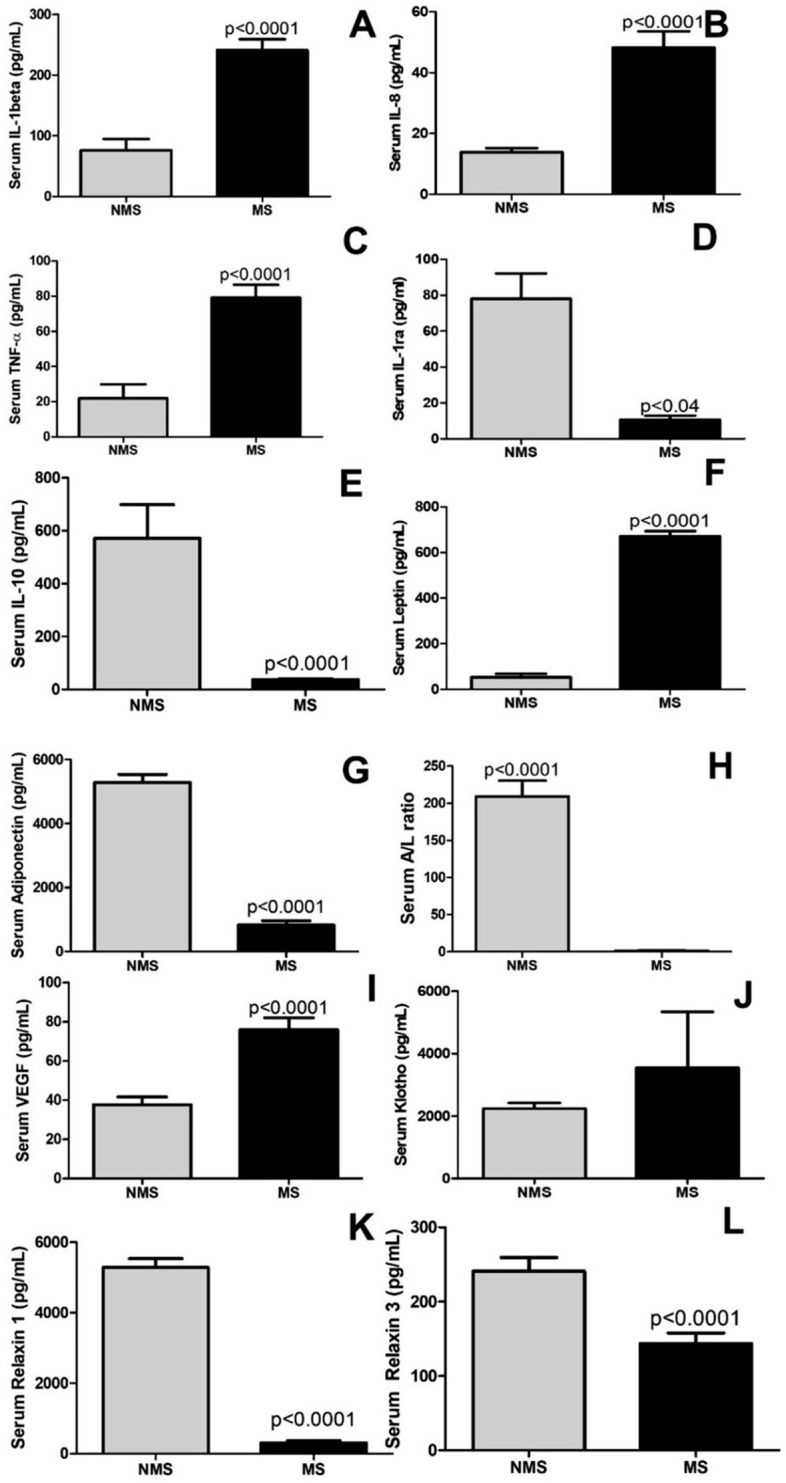
Immunological mediators in the serum in older adults with metabolic syndrome (MS) and without MS. Figure 1A (IL1-beta), Figure 1B (IL-08), Figure 1C (TNF-alfa), Figure 1D (IL1ra), Figure 1E (IL-10), Figure 1F (Leptin), Figure 1G (Adiponectin), Figure 1H (Adiponectin/ Leptin ratio), Figure 1I (VEGF), Figure 1J (Klotho), Figure 1K (Relaxin 1), Figure 1L (Relaxin 3).

Figure 1 shows the levels of circulating adiponectin, adiponectin/leptin ratio, and pro-fibrotic (VEGF), and anti-fibrotic (Klotho, Relaxin-1, Relaxin-3) factors in the serum. The results demonstrate increased levels of pro-inflammatory mediators in the participants with MS IL-1beta (*p* < 0.0001); IL-8 (*p* < 0.0001); TNF-alpha (*p* < 0.0001); leptin (*p* < 0.0001) and pro-fibrotic VEGF (*p* < 0.0001) compared to those without MS. There was a significant decrease in the levels of anti-inflammatory factors IL-1ra (*p* < 0.04); IL-10 (*p* < 0.0001); adiponectin (*p* < 0.0001); adiponectin/leptin ratio (*p* < 0.0001) and anti-fibrotic Klotho (*p* < 0.0001); relaxin-1 (*p* < 0.0001); relaxin-3 (*p* < 0.0001) in older adults with MS compared to without MS.

### Pulmonary inflammatory and fibrotic factors

**Figure 2:**
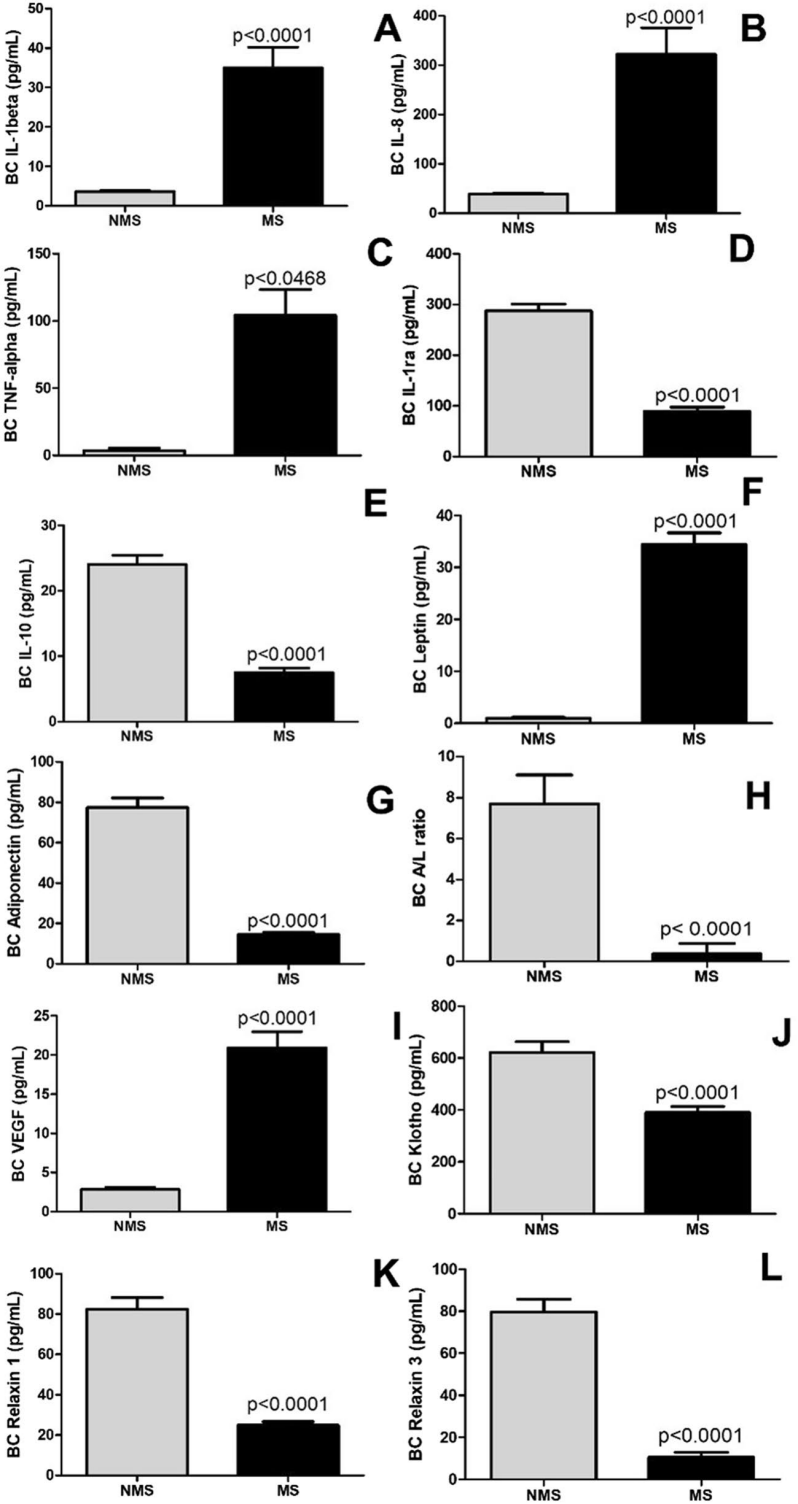
Immunological mediators in breath condensate (BC) in older adults with and without metabolic syndrome (MS). Figure 2A (IL1-beta), Figure 2B (IL-08), Figure 2C (TNF-alfa), Figure 2D (IL1ra), Figure 2E (IL-10), Figure 2F (Leptin), Figure 2H (Adiponectin), Figure 2G (Leptin/Adiponectin ratio), Figure 2I (VEGF), Figure 2 J (Klotho), Figure 2 K (Relaxin 1), Figure 2L (Relaxin 3).

Figure 2 shows the levels of pro-inflammatory factors such as IL-1beta (Fig. 2A); IL-8 (Fig. 2B); TNF-alpha (Fig. 2C); leptin (Fig. 2F), anti-inflammatory factors such as IL-1ra (Fig. 2D); IL-10 (Fig. 2E); adiponectin (Fig. 2H); adiponectin/leptin ratio (Fig. 2G), pro-fibrotic VEGF (Fig. 2I), anti-fibrotic Klotho (Fig. 2J); relaxin-1 (Fig. 2K); relaxin-3 (Fig. 2L) in breath condensate. The results demonstrated increased levels of pro-inflammatory factors leptin (*p* < 0.04); IL-1beta (*p* < 0.04); IL-8 < 0.0001; TNF-alpha (*p* < 0.0468) and pro-fibrotic VEGF (*p* < 0.0001) in breath condensate in the participants with MS compared to without MS. However, decreased levels of anti-inflammatory factors adiponectin (*p* < 0.0001); IL-1ra (*p* < 0.0001); IL-10 (*p* < 0.0001); adiponectin/leptin ratio (*p* < 0.0001) and anti-fibrotic relaxin 1 (*p* < 0.0001); relaxin 3 (*p* < 0.0001); Klotho (p < 0.0001) in breath condensate were observed in those with MS compared to without MS.

### Lung function and metabolic syndrome

**Figure 3:**
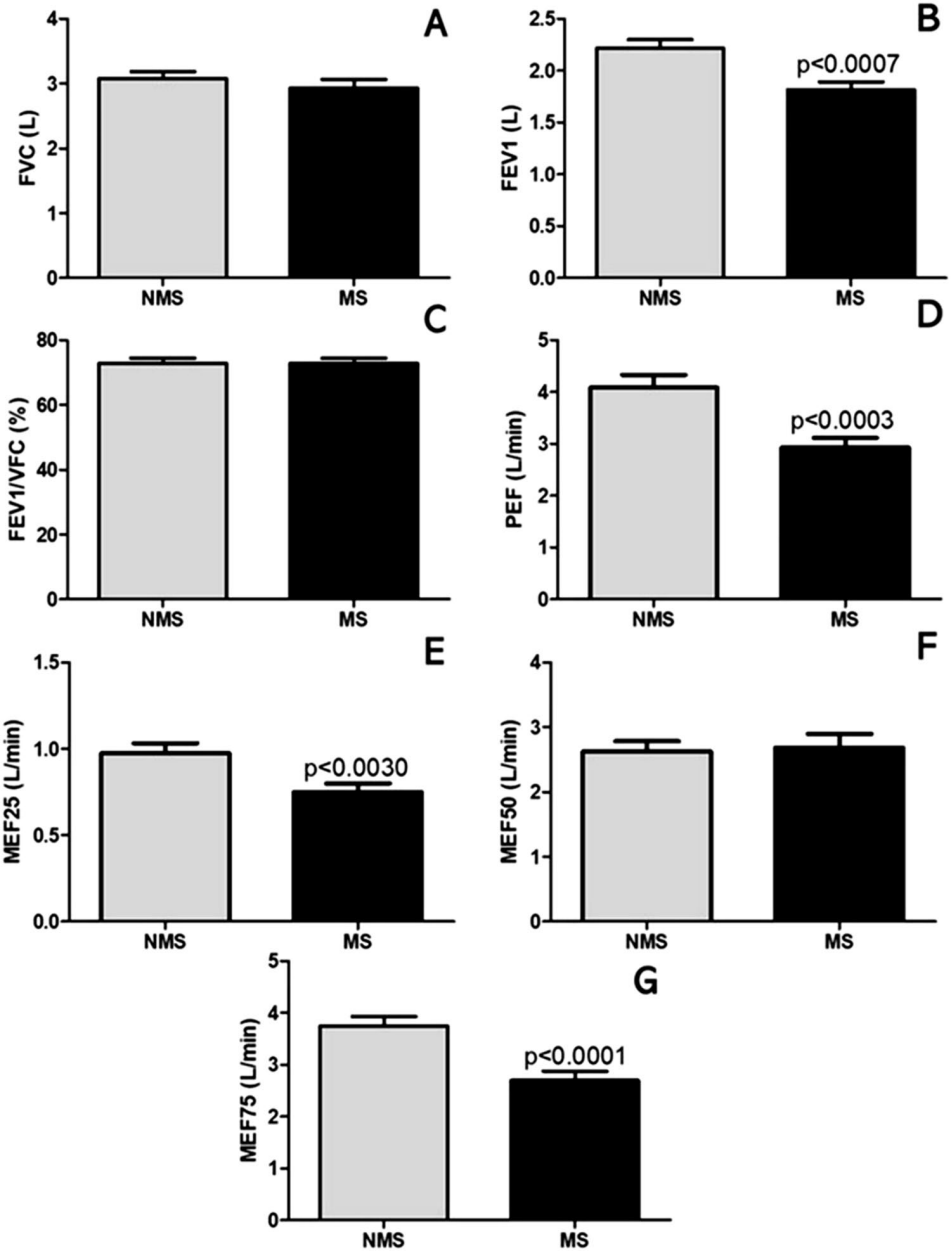
Spirometric parameters for lung function in older adults with and without metabolic syndrome (MS). Figure 3A (FVC), Figure 3B (FEV1), Figure 3C (FEV1/FVC), Figure 3D (PEF), Figure 3E (MEF25), Figure 3F (MEF50), Figure 3G (MEF75).

Figure 3 shows the lung function parameters, FVC (Fig. 3A); FEV-1 (Fig. 3B); FEV1/FVC (Fig. 3C); Peak Expiratory Flow (Fig. 3D); Maximum Expiratory Flow 25% (Fig. 3E); Maximum Expiratory Flow 50% (Fig. 3F); Maximum Expiratory Flow 75% (Fig. 3G) in MS and without MS groups. The results demonstrated reduced FEV1 (*p* < 0.0007); PEF (*p* < 0.0003); MEF25 (*p* < 0.0030); MEF75 (*p* < 0.0001) in those with MS, compared to without MS.

### Lung mechanics in metabolic syndrome

**Figure 4:**
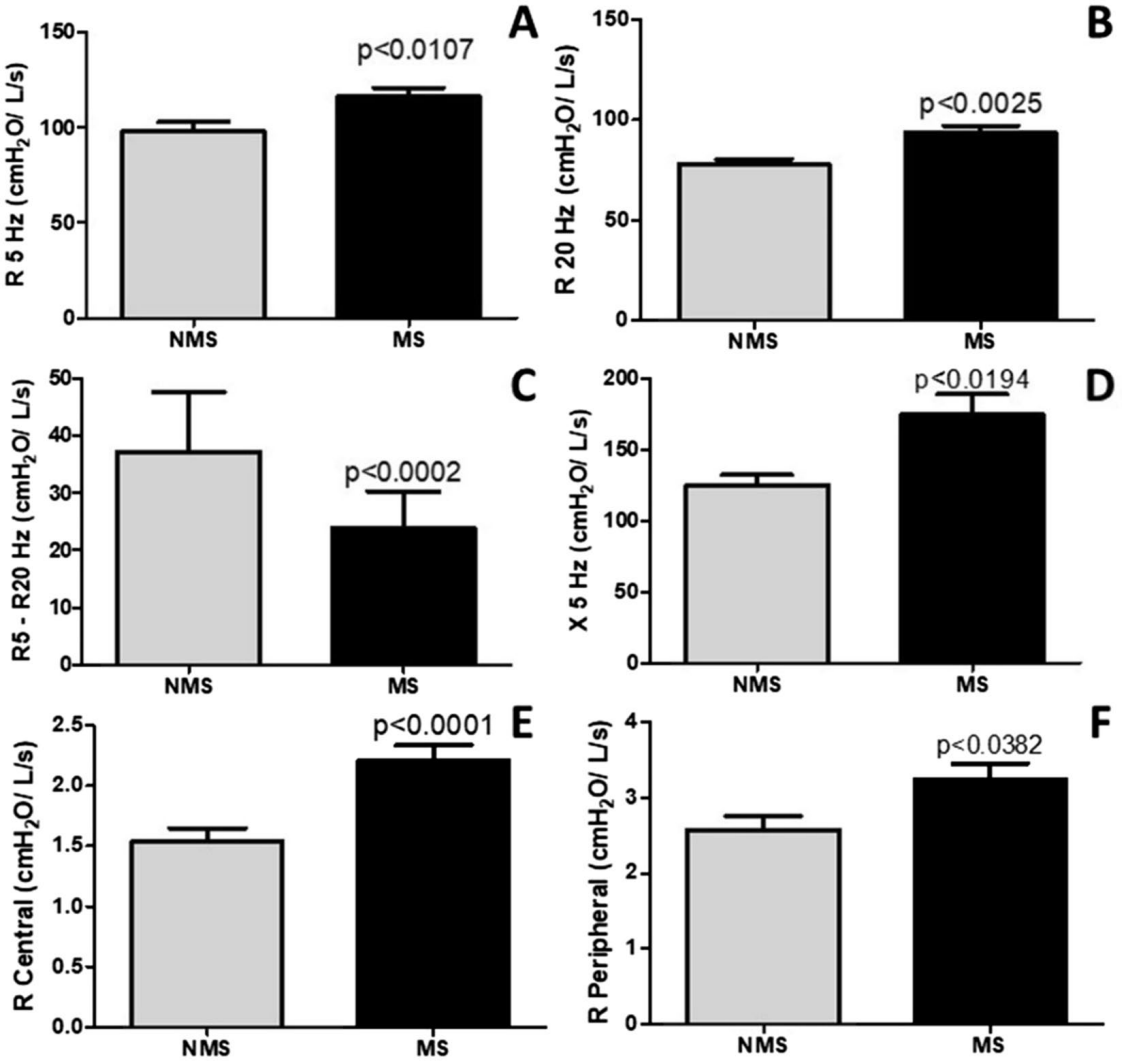
Lung mechanics evaluated by impulse oscillometry (IOS) in older adults with and without metabolic syndrome (MS). Figure 4A (R5Hz), Figure 4B (R20Hz), Figure 4C (R5Hz-R20Hz), Figure 4D (X5Hz), Figure 4E (R Central), Figure 4F (R Peripheral).

Figure 4 shows the lung mechanics parameters such as R5Hz, R20Hz, R5Hz-R20Hz, X5Hz, RCentral, and RPeripheral. The results demonstrated increased resistance of respiratory system (R5Hz; *p* < 0.0107), proximal airways (R20Hz; *p* < 0.0025), distal airways (R5Hz-R20Hz; *p* < 0.0002), tissue reactance (X5Hz; *p* < 0.0194), resistance of proximal tissue (RCentral; *p* < 0.0001) and resistance of distal tissue (RPeripheral; *p* < 0.0382) in MS compared to without MS.

### Respiratory muscle strength and hand grip strength

**Figure 5:**
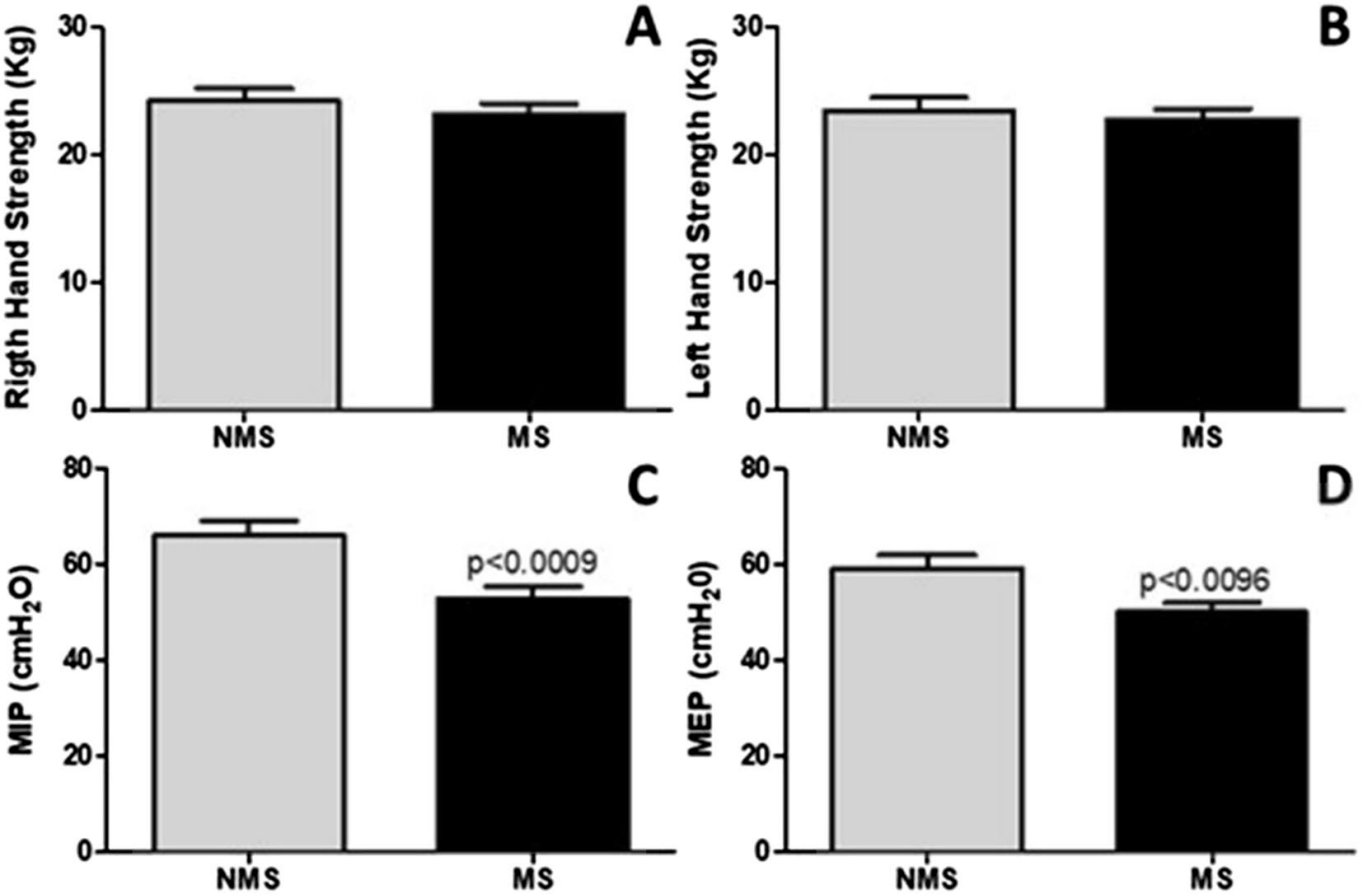
General strength measured by hand grip strength and respiratory muscle strength measured by manovacuometer in older adults with and without metabolic syndrome MS. Figure 5A (Right hand grip strength), Figure 5B (Left hand grip strength), Figure 5C (Maximal inspiratory pressure – MIP), Figure 5D (Maximal expiratory pressure – MEP).

Figure 5 shows the general strength evaluated by hand grip [Fig. 5A (right hand) and B (left hand)] and the respiratory muscle strength, evaluated by maximal inspiratory pressure (MIP) (Fig. 5C) and maximal expiratory pressure (MEP) (Fig. 5D). The results demonstrated that MS did not induce changes in general strength [(Fig. 5A; right hand; *p* > 0.05) and (Fig. 5B; left hand; *p* < 0.0009) compared with without MS group. However, respiratory muscle strength (MIP; *p* < 0.0009) and (MEP; *p* < 0.0096) in the MS group were lower compared with without MS group.

## Discussion

This study shows for the first time that the systemic low-grade inflammation commonly observed in individuals with MS is similarly present in the lungs. The results reveal that older adults with MS display impaired lung function and mechanics, and a notable increase in the resistance of whole respiratory system as well as in the airways. Furthermore, while the general strength was preserved in both MS and without MS, there was a significant deterioration in respiratory muscle strength in the older adults with MS.

The functional and structural alterations observed in the lungs (airways and parenchyma), in older adults with MS suggest that the lung may be a potential target organ in older adults in the setting of MS. The increases in airway and tissue resistance in the lungs, indicating the process of remodeling, is characterized by the accumulation of extracellular matrix proteins (collagen, elastin, proteoglycans, laminins) and reflect the structural and functional alterations in the lungs^9^. The alterations of the inflammatory milieu in the lungs are considered a causative factor in its remodeling process^9,15^.

MS leads to immune hyperactivation mainly characterized by increased levels of pro-inflammatory cytokines (i.e. IL-1beta, IL-6, IL-8, TNF-alpha, etc.), pro-inflammatory adipokine (leptin) and pro-fibrotic growth factors (i.e. VEGF, TGF-beta, etc.)^9,15^. The increased levels of proinflammatory mediators can disrupt the release of anti-inflammatory cytokines such as IL-1ra, IL-10, and anti-inflammatory adipokines such as adiponectin, accounting for impairment of the normal immune function, alter normal lung function and may perpetuate the development and progression of chronic diseases^2,9^. Previous reports have showed robust correlations of MS with structural and functional alterations in the heart^16^, blood vessels^16^ and kidneys^16^. The present study showed that concomitant with the systemic responses, there was strong pro-inflammatory and pro-fibrotic responses in the lungs of older adults with MS, with a reduced anti-inflammatory response.

Metabolic alterations and impairments are classic features of aging, and they are typically mediated by a compromised immune response leading to inflammaging and immunosenescence^17,18^. The present study highlights that MS in older adults is associated with substantially increased release of systemic pro-inflammatory mediators such as IL-1beta, IL-8, TNF-alpha, leptin, and the pro-fibrotic mediator, VEGF, when compared to older adults without MS. More importantly, the study showed for the first time that anti-inflammatory mediators such as IL-1ra, IL-10, and adiponectin, and anti-fibrotic mediators such as Klotho, Relaxin 1, Relaxin 3^19,20^ are reduced in the older adults with MS. Such effects observed in the older adults with MS may accelerate the process of senescence leading to an increase in the risk for CVD^2,21^. A previous study demonstrated that in patients with idiopathic interstitial lung diseases, reduced serum levels of klotho proteins were associated with reduced lung function^19^. This is similar to the data in the present study with reduced levels of klotho proteins in serum related to impaired lung function in older adults with MS.

The presentation of an amplified inflammatory state in the lungs and systemically, in the older adults with MS is interesting, particularly considering the lack of such data in this population. While the underlying mechanisms remain unclear, the present study showed that such pro-inflammatory and pro-fibrotic responses in the lungs were followed with increased resistance of the respiratory system (R5Hz), proximal airways (R20Hz), distal airway (R5Hz-R20Hz) and proximal (RCentral) and distal (RPeripheral) pulmonary tissue, in addition to increases in the resistance of the distal airways (X5Hz). In fact, the impairment of pulmonary mechanics observed in the present study reflects structural changes in different pulmonary compartments^22^, similar to the detrimental changes that chronic subclinical inflammation and pro-fibrotic mediators provoke in the cardiovascular system, to both structure and function^23^.

Increased levels of VEGF have been associated with different pulmonary diseases, such as asthma, chronic obstructive pulmonary disease, and idiopathic pulmonary fibrosis, with altered established fibrotic processes^24–26^. VEGF induces fibrosis via fibroblast and smooth muscle proliferation and activation, leading to synthesis and release of larger amounts of extracellular matrix proteins, such as collagen and elastic fibers, proteoglycans and laminins^24–26^. The present study revealed that older adults with MS presents higher levels of VEGF compared to those without MS, in circulation (systemic response) as well as in the breath condensate (pulmonary response). Thus, the airway obstruction found in older adults with MS could be the result of central airway remodeling due to pro-inflammatory and pro-fibrotic pulmonary processes^27,28^. For the distal airways, it can be due to a decrease in the retraction capacity of the lung tissue, induced by accumulation of elastic fibers that may result in increased lung elastance, as observed in the present study showing increases in RPeripheral values^29,30^.

Beyond the impairment of lung function and mechanics, and systemic and pulmonary immune response, the present study also showed a reduction in the respiratory muscle strength, while the general muscle strength remained stable, in older adults with MS compared to without MS. This is a clinically significant finding with respect to the impairment of lung function and mechanics, since the number of cardiorespiratory events in older adults is typically higher than in younger population. These are even at a higher magnitude in older adults with MS^31^. Likewise, during the cardiorespiratory events, the need for intubation following mechanical ventilation is higher among older adults and the impaired lung function and reduced diaphragm muscle mass may adversely affect the prognosis^32^. Thus, the reduced respiratory muscle strength in older adults with MS observed in this study should be carefully considered, particularly in critically ill older adults, as these patients are more susceptible to respiratory issues and may stay longer under mechanical ventilation.

The present study has a few limitations worth noting. The blood glucose levels were not measured, since all MS older adults were diagnosed with diabetes, but well-controlled and stable under standard medication provided by the Brazilian government for at least 24 months. While exhaled breath condensate is a generally accepted method of sampling airway secretions, the approach is not flawless and the disease itself could introduce artifacts by disrupting the endothelial-epithelial barrier. Therefore, additional measurements to further assess this disruption is warranted in future studies. The lack of computerized tomography (CT) measurement of structural changes in the lungs is another limitation and the related data should be considered with caution. Further, we did not determine the impact of each component of the MS separately in the derangement of lung structure, function, and immune response.

In conclusion, MS affects lung function and mechanics, and immunological response in older adults. Given the wide prevalence of MS in the older population, it is crucial that we understand the underlying mechanisms by which the metabolic derangements impact the lung. This will allow the development of better strategies to prevent complications. In fact, the present study highlights the clinical relevance of assessing not only systemic inflammation but also pulmonary inflammation along with lung mechanics in older adults with MS. Such an approach will enable the development of more directed therapeutic interventions to improve the outcomes in older adults with MS.
